# Prevalence and Risk Factors of Resident to Staff Aggression in Long Term Care Facilities in Hong Kong

**DOI:** 10.1093/geroni/igaf122.874

**Published:** 2025-12-31

**Authors:** Elsie Yan, Daniel W L Lai, Habib Chaudhury, Karl Pillemer, Mark Lachs

**Affiliations:** The Hong Kong Polytechnic University, Hong Kong, Hong Kong, China (People’s Republic); Hong Kong Baptist University, Hong Kong, China (People’s Republic); Simon Fraser University, Burnaby, British Columbia, Canada; Cornell University, Ithaca, New York, United States; Weill Cornell Medicine, New York, New York, United States

## Abstract

Resident-to-staff aggression (RSA) is common in long-term care facilities. It is associated with adverse physical and psychological consequences for staff, deteriorates resident-staff relationships, and greater staff turnover intention. Drawing on a sample of 703 care workers from 70 long-term care facilities, this study sought to determine the prevalence and risk factors of RSA in Hong Kong. RSA is common in this sample: 97.6% reported verbal aggression, 10.7% physical assault, 8.5% sexual violence, 13.7% annoying behaviors. Logistic regression analyses were conducted to determine factors associated with physical assaults, sexual violence, and annoying behaviors, controlling for duration (minutes) and location (common area vs resident rooms) of RSA. Physical assaults was associated with perpetrator behavioral problems (OR = 1.08, p<.001), resident male gender (OR = 2.40, p<.05), dementia (OR = 21.87, p<.001), staff lack of experience in dementia care (OR = 17.59, p<.001), need to provide dementia care (OR = 15.89, p<.01), lack of training (OR = 10.06, p<.01, and perceived insufficient training (OR = 2.97, p<.01). Sexual violence was associated with perpetrator male gender (OR = 22.51, p<.001), staff younger age (OR=.93. p<.05) and female gender (OR=.14, p<.01). Annoying behaviors was associated with perpetrator behavioral problems (OR = 1.07, p<.001), younger age (OR=.94, p<.95), male gender (OR = 3.04, p<.01), dementia (OR = 2.31, p<.01), staff female gender (OR=.32, p<.01), lack of experience in dementia care (OR = 3.56, p<.05), needs to provide dementia care (OR = 12.31, p<.01), lack of training (OR = 11.52, p<.001), and perceived insufficient training (OR = 2.52, p<.01). Addressing resident behavioral problems and providing sufficient staff training may help prevent RSA

